# Combined angiography and perfusion using radial imaging and arterial spin labeling

**DOI:** 10.1002/mrm.27366

**Published:** 2018-07-19

**Authors:** Thomas W. Okell

**Affiliations:** ^1^ Wellcome Centre for Integrative Neuroimaging, FMRIB, Nuffield Department of Clinical Neurosciences University of Oxford Oxford United Kingdom

**Keywords:** arterial spin labeling, brain blood flow, dynamic angiography, noncontrast, perfusion imaging, simultaneous acquisition

## Abstract

**Purpose:**

To demonstrate the feasibility of a novel noninvasive MRI technique for the comprehensive evaluation of blood flow to the brain: combined angiography and perfusion using radial imaging and arterial spin labeling (CAPRIA).

**Methods:**

In the CAPRIA pulse sequence, blood labeled with a pseudocontinuous arterial spin labeling pulse train is continuously imaged as it flows through the arterial tree and into the brain tissue using a golden ratio radial readout. From a single raw data set, this flexible imaging approach allows the reconstruction of both high spatial/temporal resolution angiographic images with a high undersampling factor and low spatial/temporal resolution perfusion images with a low undersampling factor. The sparse and high SNR nature of angiographic images ensures that radial undersampling artifacts are relatively benign, even when using a simple regridding image reconstruction. Pulse sequence parameters were optimized through sampling efficiency calculations and the numerical evaluation of modified pseudocontinuous arterial spin labeling signal models. A comparison was made against conventional pseudocontinuous arterial spin labeling angiographic and perfusion acquisitions.

**Results:**

2D CAPRIA data in healthy volunteers demonstrated the feasibility of this approach, with good vessel visualization in the angiographic images and clear tissue perfusion signal when reconstructed at 108‐ms and 252‐ms temporal resolution, respectively. Images were qualitatively similar to those from conventional acquisitions, but CAPRIA had significantly higher SNR efficiency (48% improvement on average, *P* = 0.02).

**Conclusion:**

The CAPRIA technique shows potential for the efficient evaluation of both macrovascular blood flow and tissue perfusion within a single scan, with potential applications in a range of cerebrovascular diseases.

## 1 INTRODUCTION

In many cerebrovascular diseases, the ability to visualize the flow of blood through the arteries is crucial for diagnosis and treatment planning. Knowledge of downstream tissue perfusion is also critical, as arterial disease may be compensated for by efficient collateral flow, or perfusion abnormalities may exist without overt macrovascular changes. Conventional invasive x‐ray angiographic techniques have high spatial and temporal resolution, but yield limited information on tissue perfusion, require exposure to ionizing radiation, and the procedure carries some risks to the patient.^1^ Contrast‐enhanced CT has the ability to obtain both angiographic and perfusion information, although radiation doses can start to become considerable, there is a risk of contrast agent reaction, spatial coverage can be limited, and temporal resolution is often low.^2^

Noninvasive alternatives that do not require ionizing radiation exposure are therefore appealing, particularly for pediatric applications and for longitudinal assessments. Gadolinium‐based contrast agents have been used with MRI to obtain both angiographic and perfusion images, but performing both acquisitions would require separate contrast agent injections and a delay between scans unless simultaneous acquisition can be performed.^3^ In addition, these contrast agents are contraindicated in some patients^4^ and there is some concern about Gadolinium retention in the brain,^5^ so a noncontrast alternative is desirable.

Arterial spin labeling (ASL) is a noncontrast MRI‐based technique that uses inverted blood water as an endogenous tracer, which has been used for both angiography^6^, ^7^, ^8^ and perfusion imaging.^9^, ^10^, ^11^ However, to obtain a complete assessment of blood supply to the brain, separate angiographic and perfusion acquisitions generally have to be performed, which may be too time‐consuming in a busy clinical setting.

In this study, a new ASL imaging method is proposed in which the blood is continuously imaged after labeling as it flows through the vascular tree and into the tissue, allowing both time‐resolved angiographic and perfusion images to be reconstructed from the same raw data set. This makes efficient use of the postlabeling delay “dead time” normally required in perfusion imaging, while also giving a greater degree of flexibility in the image reconstruction process, allowing the resulting images to be retrospectively tailored to the hemodynamics of each subject. In this proof of concept study, the principles and theoretical optimization of this approach are described, and feasibility is demonstrated using a 2D multislice implementation, although these ideas can be extended to a full 3D acquisition. Comparison is also made against conventional ASL angiographic and perfusion imaging acquisitions. This study builds on the work previously presented in abstract form.^12^

## 2 METHODS

### 2.1 Pulse sequence design

**Figure 1:**
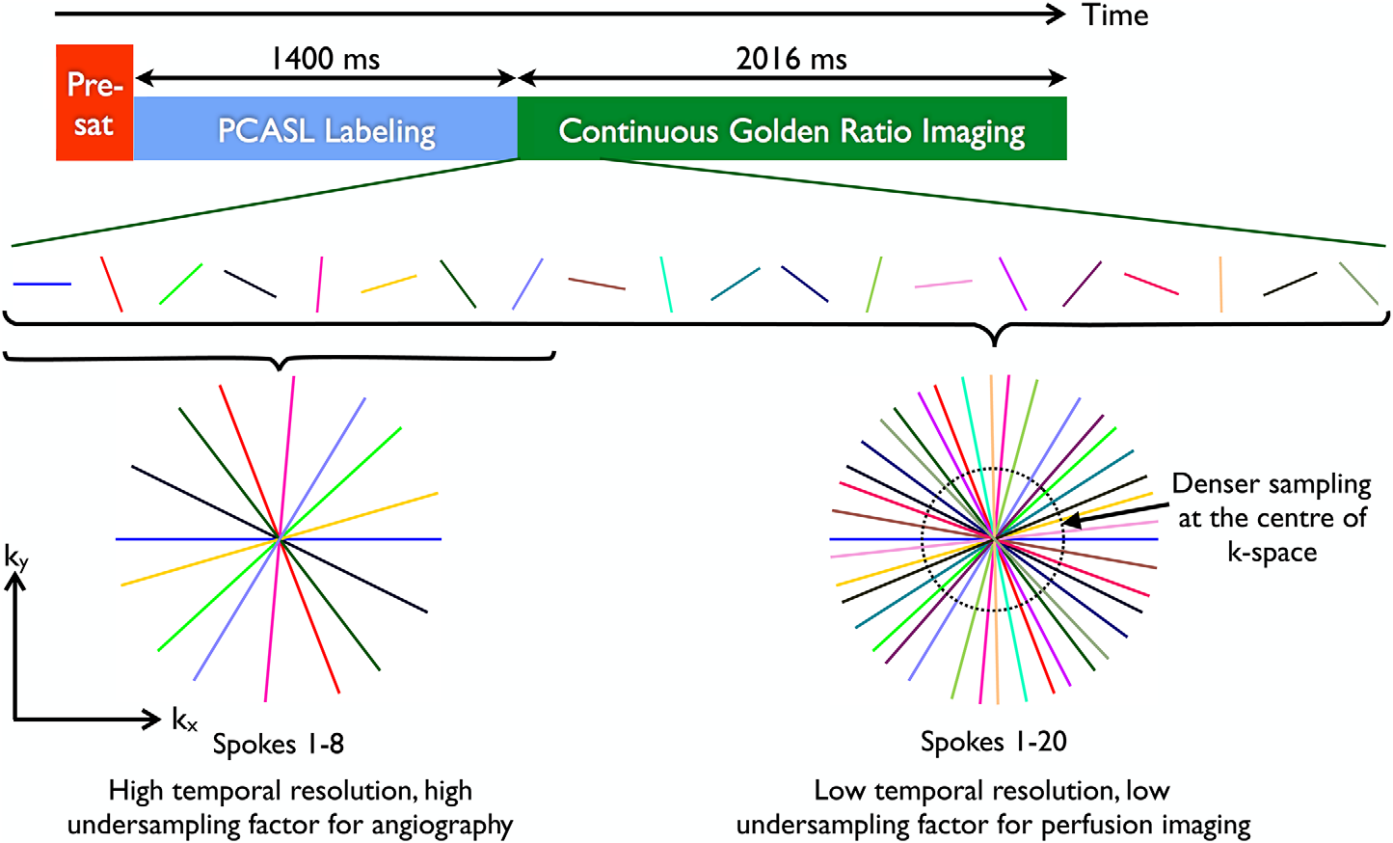
Combined angiography and perfusion using radial imaging and arterial spin labeling (CAPRIA) pulse sequence schematic. After presaturation and pseudocontinuous arterial spin labeling (PCASL) labeling, a continuous golden ratio imaging is performed for a period of 2016 ms. For angiography, high temporal resolution is desirable, so a small number of radial spokes can be extracted that corresponds to a small temporal window. This results in a high undersampling factor, but because of the sparsity and high SNR of angiographic images, this can be tolerated. For perfusion imaging, the blood flow dynamics are slower, so a larger number of radial spokes can be used, reducing the undersampling factor to better condition image reconstruction. The center of k‐space is more densely sampled with this trajectory, so lower spatial resolution images can be reconstructed with better fidelity

A schematic of the combined angiography and perfusion using radial imaging and ASL (CAPRIA) pulse sequence is shown in Figure 1. A water suppression enhanced through T_1_ effects presaturation module, as used previously,^13^, ^14^ provides background suppression, followed by a pseudocontinuous ASL (PCASL) pulse train^15^ to invert blood flowing through a defined labeling plane within the neck. Immediately after labeling, a continuous spoiled gradient‐echo radial readout scheme is used to image the blood as it flows through the arteries and into the brain tissue. One radial line, or “spoke,” in k‐space is acquired after each low flip angle excitation pulse, and the angle of each spoke is incremented by $\phi_{G}=111.25^{\circ }$ relative to the previous spoke. This golden ratio angle increment ensures that each new spoke fills the largest remaining gap present in k‐space, such that the combination of any arbitrary number of contiguously acquired spokes gives almost uniform azimuthal sampling.^16^

This sampling approach is well suited for combined angiography and perfusion imaging: Visualizing rapid blood flow through the arteries demands high spatial and temporal resolution, but the images are sparse and have high SNR due to the concentration of labeled blood within the vessels and minimal T_1_ relaxation at early time points. Thus, a small number of radial spokes can be combined to reconstruct angiographic images with high temporal resolution, but also a high undersampling factor, which results in relatively low‐intensity noise‐like artifacts for sparse data, which should not impede vessel visualization if the SNR is sufficiently high.^17^, ^18^ By the time the labeled blood has reached the tissue it is more dispersed, it occupies a much lower voxel volume fraction, and a greater degree of T_1_ decay has occurred, lowering the SNR. However, the dynamics of the signal changes are slower and lower spatial resolution can be tolerated. Therefore, perfusion images can be reconstructed using a larger number of radial spokes, giving poorer temporal resolution but a lower undersampling factor, better conditioning the image reconstruction of this low SNR, low sparsity signal. In addition, lower spatial frequencies are more densely sampled with this trajectory, so lower spatial resolution images can be reconstructed with even smaller undersampling factors. This flexibility means that time‐resolved angiographic or perfusion‐like images can be reconstructed from the same raw data set at any arbitrary time points after labeling, allowing the retrospective adaptation to the hemodynamics of the subject.

### 2.2 Sequence looping

**Figure 2:**
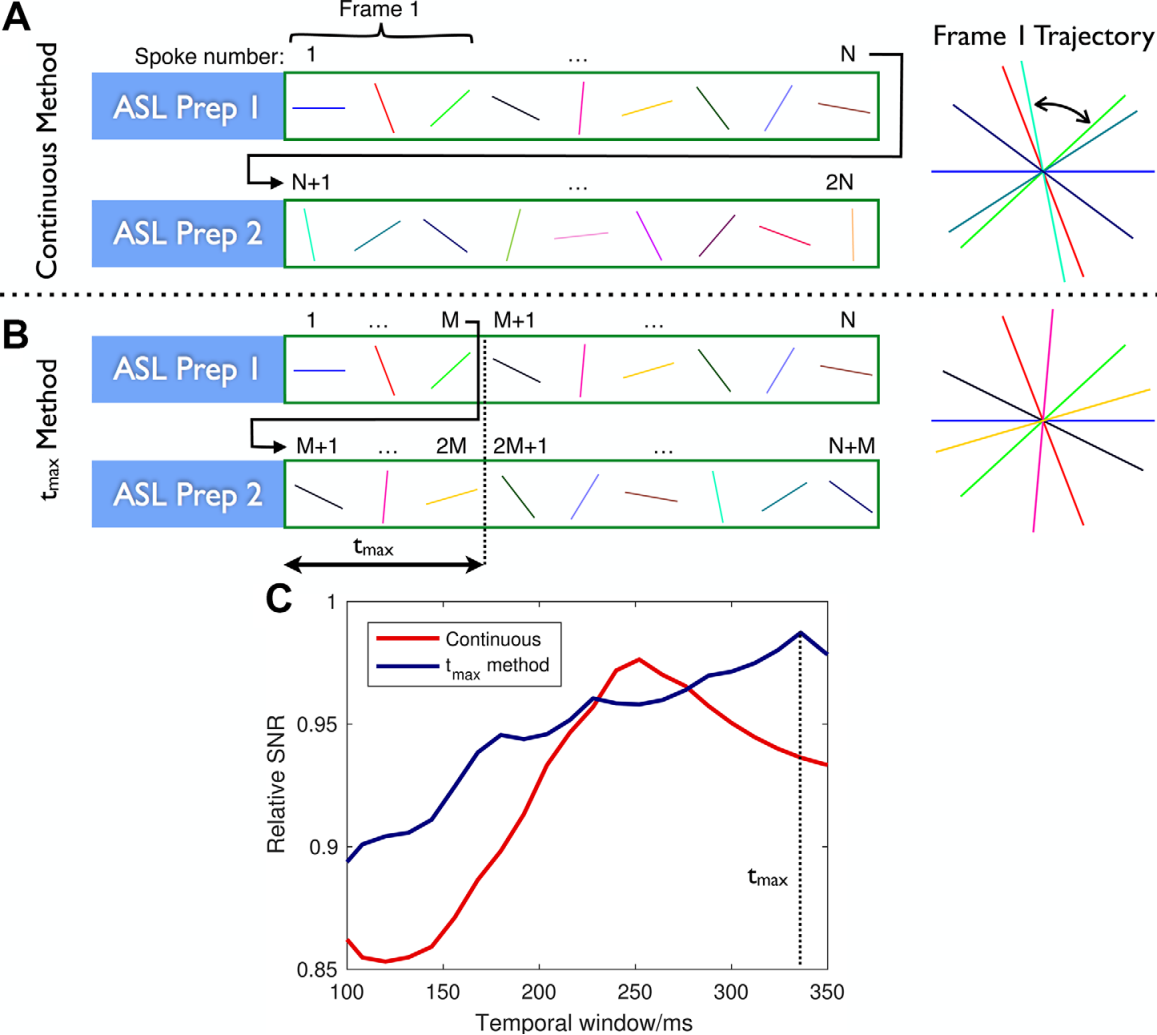
Sequence looping: Comparison of 2 methods for looping through radial spoke angles across multiple arterial spin labeling (ASL) preparations. Interleaved control scans are omitted here for clarity. In both cases, spokes 1 through *N* are acquired after the first ASL preparation. A, In the continuous method, after the second ASL preparation the first acquired spoke is *N* + 1, so no 2 spokes are ever acquired at the same azimuthal angle. B, In the *t*_max_ method, the first acquired spoke after the second ASL preparation is *M* + 1, where *M* corresponds to the number of spokes that can be acquired within the maximum temporal window, *t*_max_. As long as the reconstructed temporal window does not exceed *t*_max_, spokes within each time frame do not overlap. In this simple example, the radial spokes that would be used to reconstruct the first time frame are shown (where the temporal window has been set to *t*_max_ in this case), demonstrating the potential for suboptimal spoke separation with the continuous method (curved arrow). C, Using protocol parameters from experiments, the sampling efficiency using the *t*_max_ method is generally larger and more consistent than the continuous method for reconstructions with temporal windows in the range of 100 to 350 ms

In practice, the number of k‐space spokes that can be acquired after each ASL preparation is insufficient to reconstruct an image of acceptable quality in isolation, so data acquired over multiple ASL preparations must be combined. This raises the question of how best to choose the azimuthal angles of radial spokes acquired after subsequent labeling periods to give the most uniform coverage of k‐space when the data are combined. One choice, referred to as the “continuous” method here, involves continuing to increment the azimuthal angle across all ASL preparations, such that for the *i*th spoke acquired after the *n*th ASL preparation, the azimuthal angle, $\phi_{c}$, is given by

(1) $$\phi_{c}=(i-1+\left(n-1\right)N)\phi_{G}$$

where *N* is the number of spokes acquired after a single ASL preparation. However, when combining data across preparations within a particular temporal window, the distribution of azimuthal angles is no longer guaranteed to be close to uniform, as the spokes are not acquired contiguously (Figure 2A). This can reduce SNR and increase artifacts due to poor sampling of some regions of k‐space.

In this work, a maximum temporal window to be used for image reconstruction, *t*_max_, is defined, during which time *M* radial spokes can be acquired after each ASL preparation (*M* = *t*_max_/TR). The requirement for near‐uniform azimuthal sampling now only need apply within this temporal window. The azimuthal angle, $\phi_{t}$, is then chosen to be

(2) $$\phi_{t}=(i-1+\left(n-1\right)M)\phi_{G}$$

Using this method, radial spokes combined within a given temporal window have fewer angular increments between them compared with the continuous method, improving the azimuthal sampling uniformity (Figure 2B). This improved sampling efficiency can be quantified using a relative SNR metric,^16^ which is plotted as a function of the temporal window used for reconstruction in Figure 2C, matching other imaging parameters described subsequently.

As with all ASL techniques, 2 sets of images must be acquired: 1 in which the inflowing blood is inverted (labeled), and the other in which the blood is unperturbed (controlled), with the subtraction of the 2 removing the static tissue and leaving only the blood difference signal. In CAPRIA, the acquisition of label and control data is interleaved and acquired using an identical k‐space trajectory. This ensures that undersampling artifacts arising from static tissue are removed in the complex subtraction process, and that the effects of scanner drift and subject motion on the resulting ASL images are minimized.

### 2.3 Parameter optimization simulations

One challenge of the proposed technique is that using a series of excitation pulses after the labeling period attenuates the ASL signal, with each pulse reducing the remaining control‐label difference by a factor cos(α), where α is the excitation flip angle.^19^ Therefore, the advantages of using a high flip angle to achieve a larger signal and a short TR to achieve rapid k‐space sampling must be balanced with the need for sufficient signal to remain at later time points.

In this study, the pulse sequence parameters were optimized through numerical evaluation of ASL signal models using typical physiological parameters. For perfusion imaging, the relative signal, *S_perf_*, was calculated using the control–label magnetization difference from the Buxton model,^20^ $\delta M_{B}$, modified to include the effect of the excitation pulses in a similar manner to Günther et al^19^ as follows:

(3) $$S_{\mathrm{perf}}=\text{sin}\left(\alpha \right)\delta M_{B}{\text{cos}}^{k}\left(\alpha \right)$$

where *k* is the number of RF excitation pulses experienced by the magnetization up to the time point considered. Here it is assumed that all of the labeled blood experiences all of the excitation pulses, and *k* is approximated as a continuous function, such that

(4) $$k=\left\{\begin{array}{cc} 0 & t<t_{0}\\ \left(t-t_{0}\right)/TR & t\geq t_{0} \end{array}\right.$$

where *t*_*0*_ is the time at which imaging commences. A representative arterial transit time of 1 second was assumed for the optimization.

The angiographic signal was derived from a previously described mathematical model for PCASL angiography,^21^ with the simplifying assumptions that all labeled blood water experiences all of the imaging RF pulses, as previously, and that there is no dispersion. Here it is assumed the labeled blood water takes 0.5 seconds to travel between the labeling plane and the voxel of interest.

### 2.4 Subjects and scan protocol

To demonstrate the feasibility of the CAPRIA technique, 4 healthy volunteers (1 female, age range 25‐36) were scanned under a technical development protocol agreed by local ethics and institutional committees on a 3T Siemens Verio scanner (Siemens Healthineers, Erlangen, Germany) using a 32‐channel head coil. A short time‐of‐flight angiogram was acquired as a reference to position the ASL labeling plane^14^, ^22^ and imaging slices.

The 2D CAPRIA data were acquired in 4 10‐mm slices, with the most inferior slice positioned approximately at the level of the circle of Willis. Each slice was acquired as a separate scan taking 2.5 minutes each. The PCASL pulse train parameters were set as per previous studies,^14^, ^22^ with a labeling duration of 1400 ms. Key imaging parameters include imaging time after each ASL preparation = 2016 ms, maximum temporal window (*t*_max_) = 336 ms, matrix size = 192, nominal voxel size = 1.1 × 1.1 × 10 mm^3^, TR = 12 ms, TE = 6 ms, and flip angle = 7 °. Other parameters are detailed in Table 1.

**Table 1:** Imaging and reconstruction parameters

	Parameter	CAPRIA		Conventional	
		Angiography	Perfusion	Angiography	Perfusion
Label	Labeling duration (ms)	1400		1000	1400
	Background suppression	WET presaturation		WET presaturation	
Readout	Acquired matrix size	192		192	64
	Nominal voxel size (mm^3^)	1.1 × 1.1 × 10		1.1 × 1.1 × 10	3.4 × 3.4 × 10
	Slices	4		4	4
	Flip angle (°)	7		7	90
	TE (ms)	6		6	14
	TR (ms)	12		12	3450
	Bandwidth (Hz/Pixel)	102		102	2004
	Imaging time after labeling (ms)	2016		1080	45
	Maximum temporal window, *t*_max_ (ms)	336		—	—
	Partial Fourier factor	—		—	6/8
	Spokes/lines per ASL preparation	168		90	48
	Label/control pairs per slice	21		17	8
	Acquisition time per slice (min)	2.5		1.25	0.92
Reconstruction^b^	Temporal resolution (ms)	108	252	108	252
	Time frames	18	8	10	8
	Spatial resolution (mm^3^)	1.1 × 1.1 × 10	3.4 × 3.4 × 10^a^	1.1 × 1.1 × 10	3.4 × 3.4 × 10
	Total spokes/lines per frame	189	441	153	48
	Undersampling factor	1.6	0.23^a^	2.0	1.3

a Accounting for post hoc smoothing.

b This reconstruction was used for most of the results presented in this paper; alternative parameters are used in Supporting Information Figure S1 and Supporting Information Videos S4 and S5.

WET, water suppression enhanced through T_1_ effects.

A further 4 subjects (2 female, age range 25‐47) were also scanned to compare this CAPRIA protocol with more conventional ASL angiographic and perfusion imaging acquisitions, with the order of scans randomized across subjects. For a fair comparison, serial single‐slice protocols were used in all cases and the maximum imaging time allowed was 1.25 minutes per slice for each conventional protocol (2.5 minutes in total per slice). Spatial and temporal resolutions were set to match those achieved in the CAPRIA protocol, accounting for the post hoc spatial smoothing used for perfusion imaging (described subsequently).

For the conventional angiographic acquisition, the labeling duration and readout duration were set to 1000 ms and 1080 ms, respectively, as per a previous study.^22^ The TR and flip angle were matched to the CAPRIA acquisition to give the same rate of signal attenuation in both cases. A radial readout was used with equally spaced spoke angles, with the number of spokes set to 153, the maximum achievable in 1.25 minutes.

The conventional perfusion imaging protocol used an EPI acquisition, with readout parameters used in previous studies.^14^, ^23^ To match the postlabeling delays of the CAPRIA data, only 1 average at each delay time was possible, giving a total imaging time of 0.92 minutes per slice. This reduced imaging time was accounted for in the SNR comparison described subsequently. More detailed imaging parameters for both conventional protocols are given in Table 1.

### 2.5 Image reconstruction

The raw CAPRIA data from each acquired slice were reconstructed offline in MATLAB (MathWorks, Natick, MA) using the adjoint operator to the nonuniform fast Fourier transform,^24^, ^25^ which is akin to regridding, with sampling density compensation calculated using an iterative procedure.^26^ Coil combination was performed using the adaptive combine algorithm.^27^ For most of the results, each raw data set was reconstructed in 2 ways:

1) Angiographic images were produced by setting the reconstructed temporal resolution to 108 ms and using the full spatial resolution of the acquired data, corresponding to an undersampling factor of 1.6 (relative to the Nyquist criterion for radial sampling); and
2) Perfusion images were obtained from the same raw data set by reconstructing images with a temporal resolution of 252 ms at full spatial resolution and then using post hoc spatial smoothing with a Gaussian kernel of FWHM 3.4 mm to give in‐plane resolution comparable to typical ASL perfusion images. Once the spatial smoothing has been accounted for, this equates to an undersampling factor of 0.23. Other reconstruction parameters are listed in Table 1.

To demonstrate the flexible nature of the golden ratio readout, similar angiographic and perfusion reconstructions were also performed at a variety of other temporal resolutions: For angiography these were 60 ms and 216 ms (corresponding to undersampling factors of 2.9 and 0.8, respectively) and for perfusion imaging 132 ms and 336 ms (undersampling factors 0.44 and 0.17, respectively).

Difference images were then derived through the subtraction of label and control data, taking the real part of the complex signal after phase alignment to the control image. The first angiographic image reconstructed after the long PCASL labeling period shows most of the vascular tree filled with labeled blood, with subsequent frames showing the outflow of blood. To achieve a more intuitive visualization, the “inflow subtraction” technique was used to show inflowing rather than outflowing blood.^28^, ^29^, ^30^

The conventional ASL angiograms were reconstructed using an identical image reconstruction pipeline. The temporal resolution of 108 ms and number of spokes achievable within the available acquisition time resulted in an undersampling factor of 2.0. Conventional EPI‐based perfusion images were produced using the default vendor reconstruction, which used a sum‐of‐squares coil combination procedure. In addition, to allow a direct SNR comparison of perfusion images at the same spatial resolution, the CAPRIA perfusion reconstruction was repeated, but this time only using the central 64 samples of each radial spoke, yielding images with voxel size 3.4 × 3.4 × 10 mm^3^. No post hoc spatial smoothing was used in this case.

### 2.6 Signal‐to‐noise‐ratio efficiency comparison

To compare CAPRIA with conventional approaches, the SNR efficiency of each method was considered. A rough theoretical estimate of the relative SNR efficiency, *E*, of the 2 approaches can be made by considering the signal strength, which depends on the applied flip angle, *α*; the amount of labeled blood available, which scales with the labeling duration, τ; the amount of time spent reading out the signal of interest, *t*_*RO*_; the $T_{2}^{*}$ signal decay within the TE; and the total acquisition time dedicated to that modality, *T*:

(5) $$\frac{E_{\mathrm{CAPRIA}}}{E_{\mathrm{Conv}}}=\frac{\sin\alpha_{\mathrm{CAPRIA}}\tau_{\mathrm{CAPRIA}}\sqrt{t_{RO,CAPRIA}}e^{-TE_{\mathrm{CAPRIA}}/T2^{*}}}{\sin\alpha_{\mathrm{Conv}}\tau_{\mathrm{Conv}}\sqrt{t_{RO,Conv}}e^{-TE_{\mathrm{Conv}}/T2^{*}}}\sqrt{\frac{T_{\mathrm{Conv}}}{T_{\mathrm{CAPRIA}}}}$$

where “Conv” indicates parameters from the conventional protocol. Here it was assumed that the images are reconstructed at the same spatial resolution and $T_{2}^{*}$ is taken to be that of gray matter at 3 T (35 ms). In addition, t_RO,CAPRIA_ must account for the fact that only the central portion of k‐space is used for the reconstruction of perfusion images. The additional signal attenuation caused by blood experiencing multiple RF pulses after arriving in the slice for CAPRIA is neglected due to its rather complex dependence on blood arrival time in single‐slice imaging,^21^ meaning that this expression (Equation (5)) will likely overestimate the SNR efficiency ratio.

The SNR efficiency of each experimental data set was also evaluated. The low spatial resolution CAPRIA perfusion images were used for this purpose to ensure exactly matched voxel sizes. This was done by first calculating the temporal mean image, using the time points common to both CAPRIA and conventional protocols. Next, a signal mask was derived: For angiographic images it was found that the major vessels were identified by thresholding the temporal mean image at 0.9 times the 99th‐percentile signal intensity. Because the CAPRIA and conventional angiograms were acquired and reconstructed in an identical manner, the masks derived from each data set could be combined. To avoid bias, only voxels present in both masks were included in the final signal mask. However, for perfusion imaging, the distortion and dropout artifacts present in the EPI data made the production of a common signal mask more difficult. Therefore, the signal mask was taken to be all the brain tissue present in the acquired slices, as derived from a brain extraction algorithm^31^ run on each data set separately. The background mask was a 34 × 34 mm^2^ square in the corner of each image, running through all of the slices, away from the head and any obvious artifacts.

The SNR was calculated as the mean intensity within the signal mask divided by the SD of the signal within the background mask. Because of the noise rectification in sum‐of‐squares reconstructions, the conventional perfusion SNR values were corrected by a factor of 0.65 to compensate for the underestimated noise SD in the background. To account for the differing acquisition times of each protocol, the SNR efficiency was calculated as the SNR divided by the square root of the acquisition time (nominally split equally between angiography and perfusion imaging for CAPRIA).

The estimated noise SD will include contributions from noise‐like aliased signal in the undersampled radial protocols. Although this is not *true* noise, it will likely have a similar effect on the ability to visualize vessels with low signal intensity, so it is therefore included in this calculation.

## 3 RESULTS

**Figure 3:**
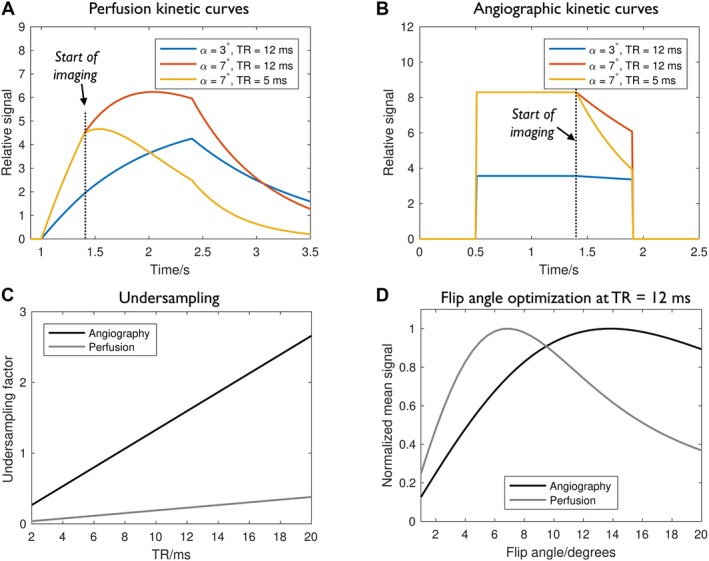
Protocol optimization: Example of the perfusion (A) and angiographic (B) signal evolution for some example combinations of flip angle (α) and TR. Imaging starts just after the end of the PCASL pulse train (at 1.4 seconds). For a small flip angle and long TR the perturbation from the imaging pulses is minimal and the perfusion signal closely follows the familiar Buxton model.^20^ Increasing the flip angle increases not only the measured signal but also the rate of signal attenuation, which is further exacerbated when the TR is reduced. The effect on the angiographic signal is similar but the attenuation effect of the imaging pulses is less severe, as there is no accumulation of labeled blood water in each voxel. Another consideration is that longer TRs lead to increased undersampling factors for a fixed scan time (C), particularly for angiography that uses higher spatial and temporal resolution. At TR = 12 ms the flip angle that maximizes the mean perfusion signal across all imaging time points occurs at 7 °, whereas it is at 14 ° for angiography (D)

The results of numerical simulations shown in Figure 3 demonstrate the need to balance rapid k‐space sampling (short TR) and high initial signal strength (high flip angle) with minimal ASL signal attenuation (low flip angle, long TR). This is particularly apparent in the perfusion signal, in which an accumulation of labeled blood water is required to produce a large peak signal. In addition, the chosen TR affects the resulting undersampling factor, but in a different way for the angiographic and perfusion reconstructions with their differing spatial and temporal resolutions. Based on these simulations, the TR was chosen to be 12 ms for this study to yield undersampling factors of 1.6 and 0.23 for angiography and perfusion imaging, respectively, within a 2.5‐minute scan time per slice. Modest undersampling should be tolerable for the sparse and high SNR angiographic data, and a perfusion undersampling factor of 0.23 is approximately equivalent to collecting 4 averages, helping to boost the SNR of this weaker signal. At this TR, the mean perfusion signal within the imaging period is maximized using a flip angle of 7 °, whereas the angiographic signal is maximized at 14 °. Due to the weaker nature of the perfusion signal and greater sensitivity to signal attenuation, a flip angle of 7 ° was chosen for the remainder of this study to optimize perfusion image quality.

**Figure 4:**
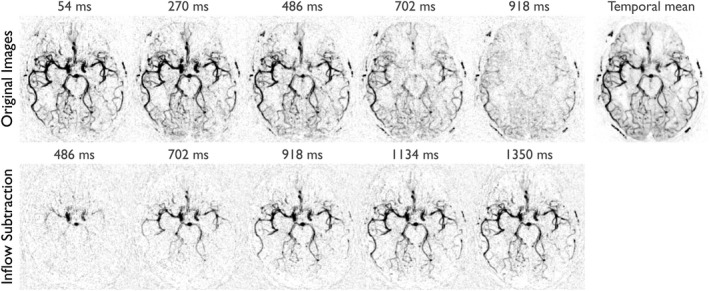
Selected frames from the CAPRIA angiogram of 1 subject reconstructed at 108 ms temporal resolution after maximum intensity projection, with inverted contrast. Images are shown with and without inflow subtraction along with the temporal mean. Times displayed are relative to the start of imaging

Example CAPRIA dynamic angiographic images from 1 volunteer are given in Figure 4 and Supporting Information Videos S1 and S2, shown as a transverse maximum intensity projection across the 4 slices for both the original reconstructed images and the inflow subtraction. Clear visualization of the passage of the blood through the arterial tree is apparent, including smaller distal vessels, and a clean subtraction of the static tissue signal was achieved. The inflow subtracted images give a more intuitive visualization of blood flowing into the arterial tree at a cost of reduced apparent SNR and distal vessel appearance. Some minor radial undersampling (“streaking”) artifacts are apparent, but these do not greatly impede image quality.

**Figure 5:**
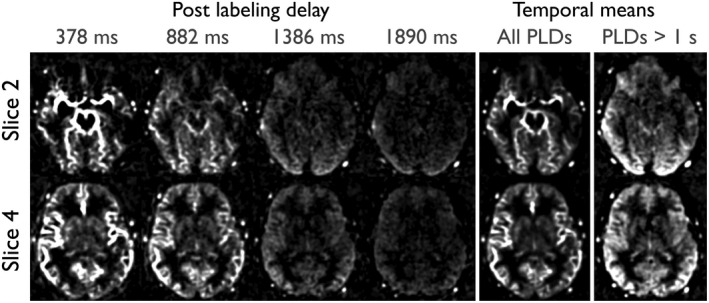
Selected frames from CAPRIA perfusion images in 2 slices reconstructed using the same raw data as Figure 4, but with a broader temporal window (252 ms) and post hoc spatial smoothing. Temporal mean images are also shown, both across all postlabeling delays (PLDs) and only for PLDs greater than 1 second, when most of the labeled blood has reached the tissue

Using the same raw k‐space data, time‐resolved perfusion images were also reconstructed, as shown in Figure 5 and Supporting Information Video S3. Tissue perfusion is clearly depicted across all of the acquired slices, with the expected pattern of macrovascular signal at early time points and broader perfusion of all brain regions at later time points. Streaking artifacts are not evident in this data due to the oversampling achievable using a lower temporal resolution and spatial smoothing.

The flexible nature of the golden ratio imaging approach is demonstrated in Supporting Information Figure S1 and Supporting Information Videos S4 and S5, where angiographic and perfusion images are reconstructed at a range of temporal resolutions. Using a smaller temporal window for reconstruction allows the temporal dynamics to be resolved more clearly but requires a greater undersampling factor and therefore increased noise‐like artifacts and lower SNR. This tradeoff can be made retrospectively according to the desired information for a particular subject.

Comparable image quality was obtained in the other 3 subjects scanned for the first part of this study, as shown in Supporting Information Figure S2.

**Figure 6:**
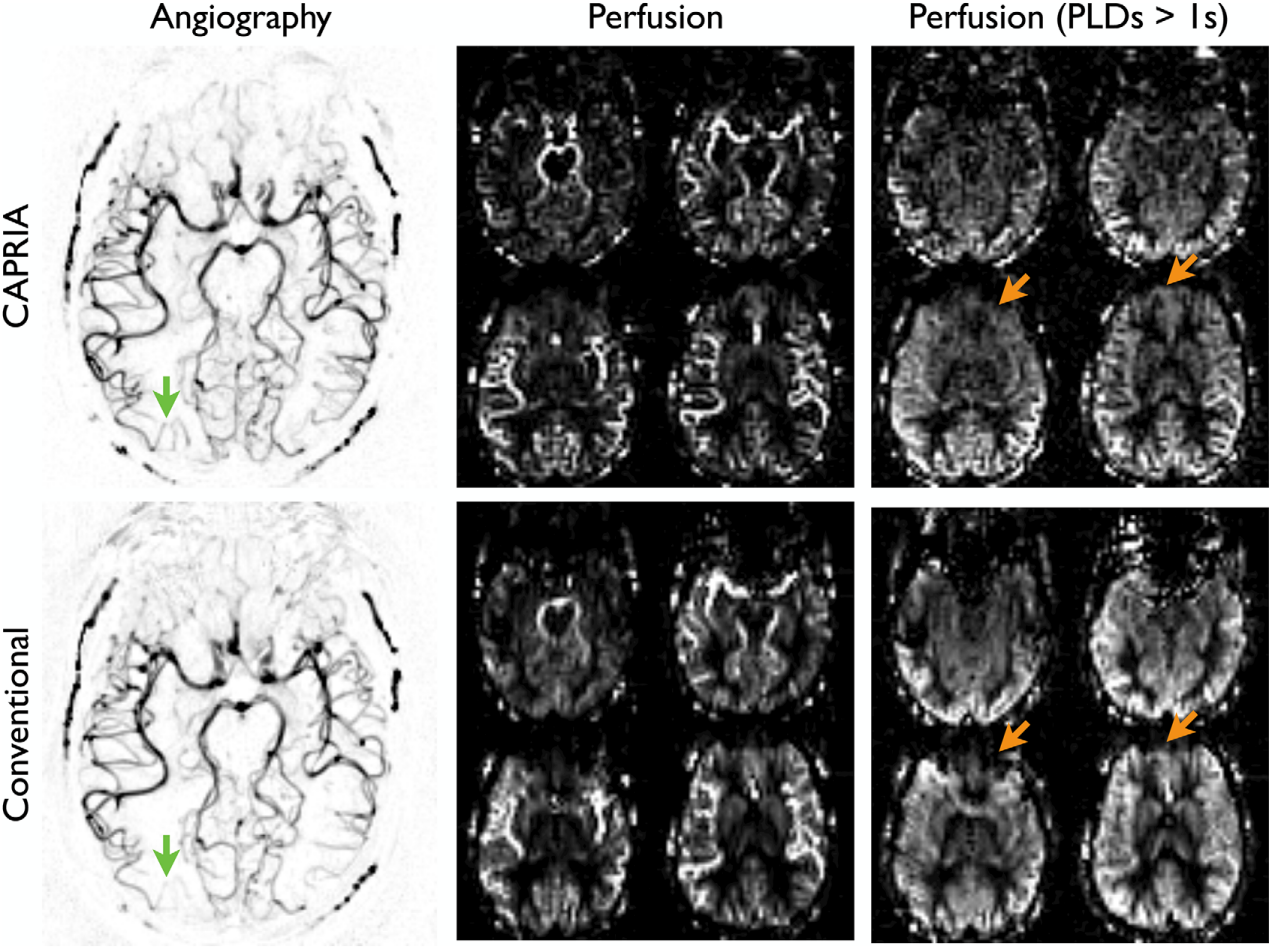
Comparison of CAPRIA data with conventional methods. Example angiographic maximum intensity projection and perfusion temporal mean images from a representative subject are shown, including perfusion images averaged over PLDs greater than 1 second, demonstrating the qualitative similarity between the methods. Reduced streaking artifacts and improved distal vessel visibility (green arrows) are apparent in the CAPRIA angiogram. The CAPRIA perfusion images (shown here reconstructed at matched spatial resolution) do not suffer from distortion and dropout artifacts visible in the EPI‐based perfusion data (orange arrows)

Example angiograms and perfusion images acquired with CAPRIA and conventional methods in the same subject are shown in Figure 6. The qualitative similarity of the images demonstrates that CAPRIA is capable of visualizing blood flow through the arteries and within the brain tissue in a manner comparable to standard approaches. The additional efficiency gained by acquiring both data sets in a single acquisition allowed the use of a lower undersampling factor for the CAPRIA angiographic reconstruction, reducing the associated streaking artifacts. In addition, the use of a longer labeling duration in CAPRIA than is typically used in ASL angiography also improved the delineation of small distal vessels. Dropout and distortion artifacts, evident in the conventional ASL perfusion images acquired with an EPI readout, are absent in the CAPRIA data.

**Figure 7:**
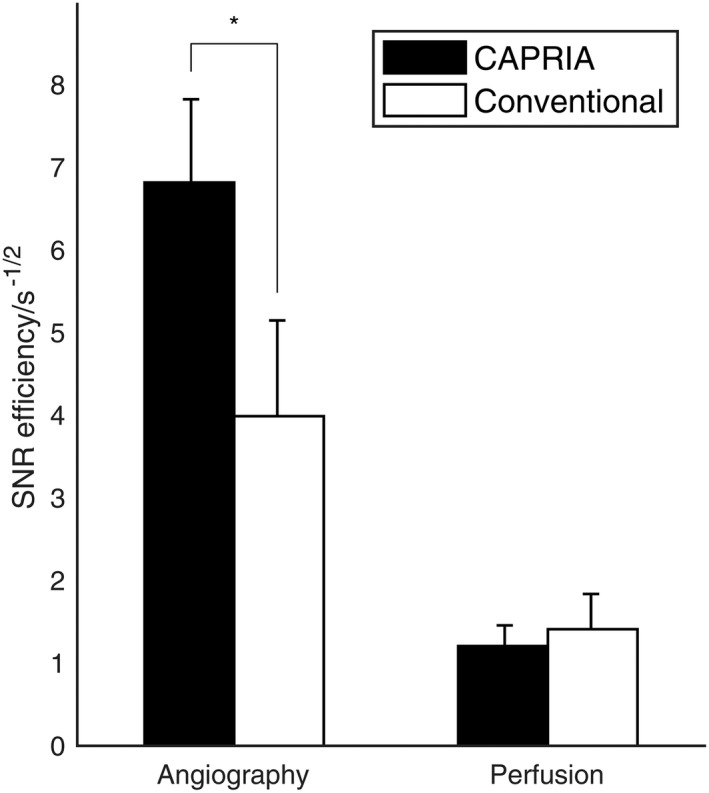
The SNR efficiency of CAPRIA and conventional methods, shown as the mean and SD across subjects. For angiography, CAPRIA has significantly higher SNR efficiency (*P = 0.005), whereas for perfusion imaging there is no significant difference

The results of the SNR efficiency comparison are shown in Figure 7. The SNR efficiency of CAPRIA angiograms was significantly higher than that of the conventional angiograms (*P* = 0.005, paired t‐test), but there was no significant difference between CAPRIA perfusion images and conventional perfusion images. The average SNR efficiency across angiographic and perfusion acquisitions combined was 48% higher for CAPRIA than for the conventional acquisitions (*P* = 0.02). These results are consistent with theoretical calculations performed using Equation (5) and the protocol parameters from Table 1. For angiography, the predicted SNR efficiency ratio was 1.6, driven by the reduced undersampling achievable with CAPRIA and longer labeling duration, matching the measured SNR efficiency ratio of 1.7 ± 0.3. For perfusion imaging, even allowing for only the central portion of k‐space being used in the reconstruction, the CAPRIA readout time, *t*_*RO,CAPRIA*_, was 60 times larger than that of the conventional EPI readout, *t*_*RO,Conv*_. This, combined with the shorter TE of CAPRIA, compensates for the lower flip angle to give a predicted SNR efficiency ratio of 1.02, consistent with lack of a significant difference found using the experimental data.

## 4 DISCUSSION

In this study, the feasibility of the CAPRIA technique for producing time‐resolved angiograms and images of tissue perfusion within a single scan was demonstrated. Furthermore, it was shown that CAPRIA produces qualitatively similar images, but with improved SNR efficiency, compared with separate conventional acquisitions. The golden ratio radial imaging approach^16^ is ideal for this application, as it allows high temporal and spatial resolution angiograms with a high undersampling factor, and lower temporal and spatial resolution perfusion images with a low undersampling factor to be reconstructed from the same raw data set. This flexibility also allows the retrospective adaptation to the hemodynamics of the subject. For example, in cases of collateral flow where blood travels along a circuitous route to reach the tissue, angiographic images can be reconstructed at later time points to visualize the collateral vessels, in a similar manner to the visualization of delayed blood arrival in extracranial vessels seen in Figure 4 and Supporting Information Video S1. Rapid shunting of blood through an arteriovenous malformation might also be better evaluated by retrospectively choosing a higher temporal resolution for reconstruction, a capability demonstrated in Supporting Information Figure S1. No a priori assumptions about the timing of blood flow are required, other than the choice of the imaging duration after each ASL preparation.

Additional benefits of this approach include the time‐resolved nature of the derived angiographic and perfusion images, which should allow the fitting of kinetic models^20^, ^21^ to extract physiological parameters, such as tissue perfusion and arterial transit time, as well as separation of potentially confounding macrovascular signal,^32^ once the signal attenuation due to the imaging pulses has been accounted for. The rapid acquisition of only a single k‐space line after each excitation pulse also makes this technique robust to signal dropout and distortion artifacts commonly seen in ASL perfusion imaging (Figure 6), as others have also observed.^33^, ^34^ The interleaving of label and control data helps to minimize subtraction artifacts,^22^ and the frequent sampling of the center of k‐space with a radial trajectory, should also make CAPRIA more robust to motion artifacts,^35^ although further work is required to verify this.

The imaging parameters used in this study were optimized through numerical simulations of the ASL signal. The tradeoff between TR, flip angle, and undersampling factor is quite complex, as it depends on the degree of undersampling that can be tolerated, which will in turn depend on the imaging time available and the image reconstruction technique. In addition, the use of a longer TR will also decrease the readout bandwidth and increase the TE, if the time between RF pulses is to be used efficiently, which has implications for SNR and flow‐induced dephasing artifacts. The ASL models used here assumed that all of the blood experiences all the RF excitation pulses, which is an oversimplification for 2D imaging, and physiological parameters typical of healthy volunteers were assumed, which may not be appropriate for patient studies. For example, if blood arrival is delayed, such that the arterial transit time to the tissue is 2 seconds instead 1 second, as assumed previously, the optimal flip angle for the perfusion signal drops from 7 ° to 5 °. Therefore, further protocol optimization may be required in future work.

There are 2 options for reconstructing perfusion images at lower spatial resolution from CAPRIA data to boost SNR: For most of this study the images were reconstructed at full spatial resolution, and spatial smoothing was used in postprocessing to achieve a lower effective voxel size. An alternative approach, used in the SNR comparison, is to directly reconstruct images using only the low spatial frequency k‐space data. The former method effectively downweights, rather than completely removes, information at high spatial frequencies, which may have some marginal benefit in terms of using the available signal, although the latter method results in reduced computational requirements for the reconstruction. However, the images produced by both approaches are broadly similar, so this choice does not appear to be critical.

The main limitation of the CAPRIA approach is the repetitive use of excitation pulses, which attenuates the ASL signal, as discussed in the context of other time‐resolved ASL imaging approaches.^19^, ^30^ This restricts the flip angle and therefore the signal strength that can be achieved compared with single‐shot readout approaches like EPI, although this is counterbalanced by the ability to sample the signal over a longer period. Both theoretical and experimental calculations demonstrated that, for the protocols used in this study, CAPRIA results in a much higher SNR efficiency for angiography and comparable SNR efficiency for perfusion imaging. Therefore, the overall SNR efficiency of CAPRIA is higher than that of separate conventional acquisitions. In future work it should be possible to trade off the SNR between angiographic and perfusion images by using a variable flip angle approach, as proposed previously to improve signal strength in later time frames for ASL angiography.^36^, ^37^

The serial 2D implementation presented here is unlikely to be of broad clinical utility due to its limited coverage and thick slices. It also necessitated comparison against serial single‐slice conventional acquisitions to isolate differences due to the proposed imaging approach alone. However, CAPRIA is generalizable to 3D imaging using an extension to the golden ratio technique,^38^ or stack‐of‐stars approach,^39^ which will be explored in future work, allowing comparison against more commonly used multislice or 3D whole‐brain conventional ASL approaches.

The CAPRIA method has similarities with previous ASL methods that use a series of low‐flip angle readouts to obtain time‐resolved images of perfusion.^19^, ^40^ Although these allow the signal evolution of blood flow to be measured, the spatial resolution would not be sufficient for angiography and the use of conventional readout approaches means the temporal resolution is fixed. The flexible nature of golden ratio radial imaging has also been used in the context of time‐resolved ASL angiography.^39^ Although CAPRIA builds on this previous work, the key difference is the use of both the flexible temporal resolution and the increased sampling density in the center of k‐space, along with appropriate protocol optimization to ensure sufficient signal remains at later time points, to allow the reconstruction of high spatial/temporal resolution angiograms and low spatial/temporal resolution perfusion images from the same raw data set.

An interesting alternative approach to CAPRIA has recently been proposed in which a time‐encoded ASL preparation is combined with 2 discrete imaging modules, one optimized for angiographic imaging and the other for perfusion imaging,^41^ allowing both sets of images to be obtained from a single scan. The time‐encoded preparation has the advantage of generating temporal information separately from the readout, reducing the number of excitation pulses required and thus limiting the ASL signal attenuation. In addition, the use of separate readout modules for angiography and perfusion imaging allows the independent optimization of imaging parameters for each. However, the use of time‐encoding intrinsically links the temporal resolution to the amount of ASL signal available by restricting the length of each time‐encoded block. In addition, the timing of all images relative to the ASL labeling must be decided in advance, which is not the case for CAPRIA. The relative merits of these 2 approaches and the potential for combining elements of both will be explored in the future.

There are a number of other refinements that could further improve upon the CAPRIA technique, including improved background suppression^42^ and intrinsic motion correction.^43^ A 3D implementation of CAPRIA will allow the signal attenuation caused by the RF excitation pulses to be accurately accounted for in modeling and potentially direct calibration of the blood equilibrium magnetization using voxels entirely within arteries, enabling absolute quantification of physiological parameters from both the angiographic^21^, ^44^ and perfusion^20^ images. In addition, the ability to capture both angiographic and perfusion information simultaneously presents the opportunity to combine this information, such as by using the angiograms to better inform the fitting of macrovascular signals in tissue perfusion estimation,^32^ or by measuring the degree of bolus dispersion within the large arteries and using this to improve perfusion quantification through modeling^45^, ^46^, ^47^ or a model‐free approach.^40^ Improved image reconstruction could be achieved through parallel imaging^48^, ^49^ and by leveraging the sparsity of the angiographic images with compressed sensing.^50^ The hope is that, after further development, CAPRIA will prove to be a useful tool for the comprehensive evaluation of brain blood flow in diseases such as atherosclerosis, acute stroke, and arteriovenous malformation.

## CONFLICT OF INTEREST

This work is the subject of a US patent application on which Thomas Okell is the sole author.

## Supporting information


**FIGURE S1** CAPRIA images reconstructed at different temporal resolutions from the same raw data as Figure 4. One frame from each reconstruction is enlarged to highlight the improved image quality that can be achieved using a wider temporal window for reconstruction, at a cost of temporal fidelity. For clarity, angiographic images are only shown up to 900 ms from the start of imaging. Angiographic reconstructions at 60 ms, 108 ms, and 216 ms correspond to undersampling factors 2.9, 1.6 and 0.8, respectively. Perfusion reconstructions at 132 ms, 252 ms, and 336 ms correspond to undersampling factors 0.44, 0.23 and 0.17, respectively.
**FIGURE S2** Temporal mean angiographic transverse MIPs and perfusion images with PLDs greater than 1 second for the other 3 subjects scanned in the first part of this study.

**VIDEO S1** Time‐resolved angiogram corresponding to the data shown in Figure 4.

**VIDEO S2** Time‐resolved angiogram corresponding to the data shown in Figure 4, with inflow subtraction.

**VIDEO S3** Time‐resolved perfusion maps, corresponding to the images in Figure 5.

**VIDEO S4** Comparison of time‐resolved angiograms reconstructed at 3 different temporal resolutions (Supporting Information Figure S1): 60 ms (left), 108 ms (middle), and 216 ms (right), corresponding to undersampling factors 2.9, 1.6 and 0.8, respectively.

**VIDEO S5** Comparison of time‐resolved perfusion maps reconstructed at 3 different temporal resolutions (as shown in Supporting Information Figure S1): 132 ms (left), 252 ms (middle), and 336 ms (right), corresponding to undersampling factors 0.44, 0.23 and 0.17, respectively.
