# Supplementary material for: Combined angiography and perfusion using radial imaging and arterial spin labeling
Source: Magn Reson Med. 2018 Jul 19;81(1):182–94. doi: 10.1002/mrm.27366 (PMC6282709; doi:10.1002/mrm.27366)
Supplement: Supplementary file 1 — FIGURE S1 CAPRIA images reconstructed at different temporal resolutions from the same raw data as Figure 4. One frame from each reconstruction is enlarged to highlight the improved image quality that can be achieved using a wider temporal window for reconstruction, at a cost of temporal fidelity. For clarity, angiographic images are only shown up to 900 ms from the start of imaging. Angiographic reconstructions at 60 ms, 108 ms, and 216 ms correspond to undersampling factors 2.9, 1.6 and 0.8, respectively. Perfusion reconstructions at 132 ms, 252 ms, and 336 ms correspond to undersampling factors 0.44, 0.23 and 0.17, respectively. FIGURE S2 Temporal mean angiographic transverse MIPs and perfusion images with PLDs greater than 1 second for the other 3 subjects scanned in the first part of this study. [file MRM-81-182-s001.pdf]

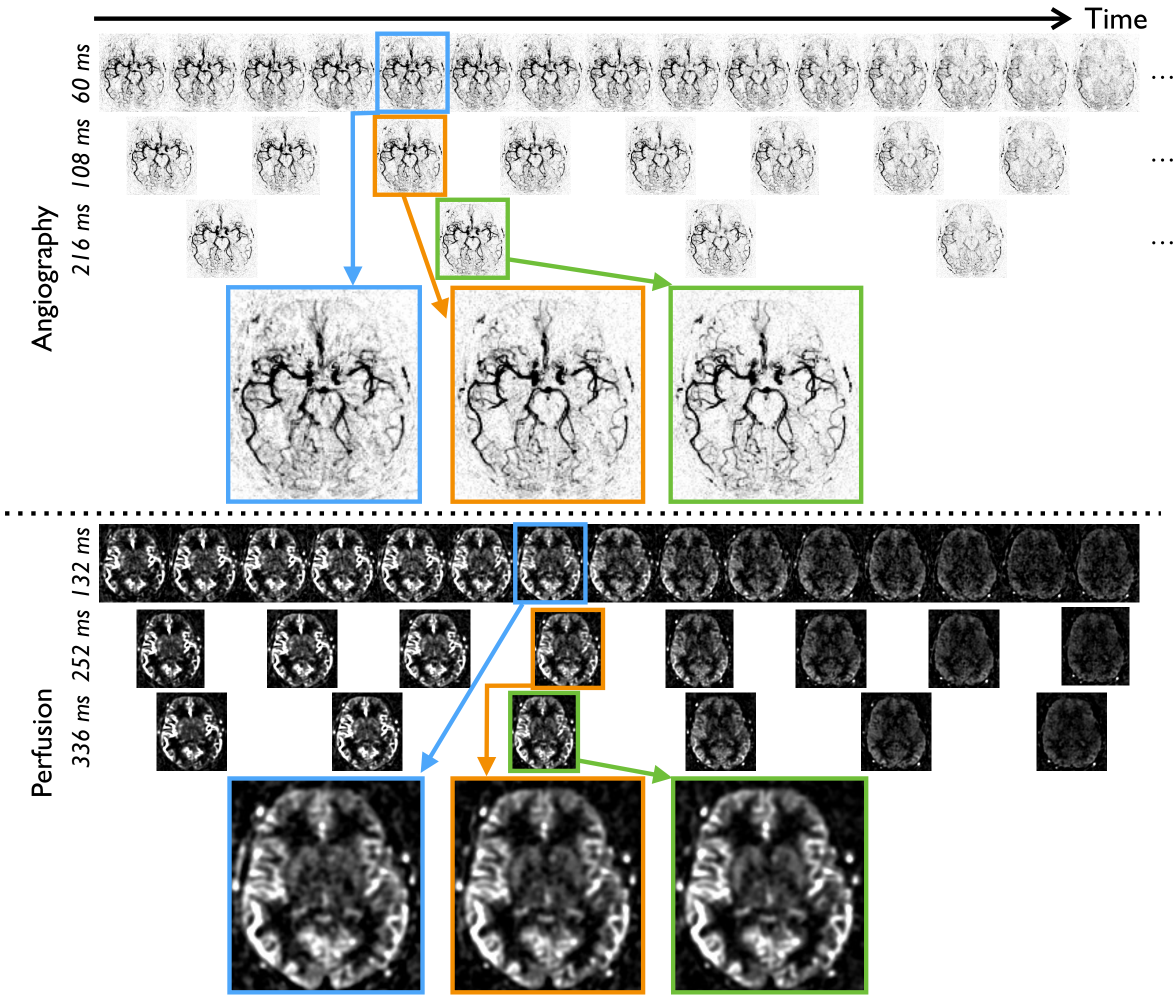

Supporting Figure S1: CAPRIA images reconstructed at different temporal resolutions from the same raw data as Figure 4. One frame from each reconstruction is enlarged to highlight the improved image quality that can be achieved using a wider temporal window for reconstruction, at a cost of temporal fidelity. For clarity, angiographic images are only shown up to 900 ms from the start of imaging. Angiographic reconstructions at 60 ms, 108 ms and 216 ms correspond to undersampling factors 2.9, 1.6 and 0.8, respectively. Perfusion reconstructions at 132 ms, 252 ms and 336 ms correspond to undersampling factors 0.44, 0.23 and 0.17, respectively.

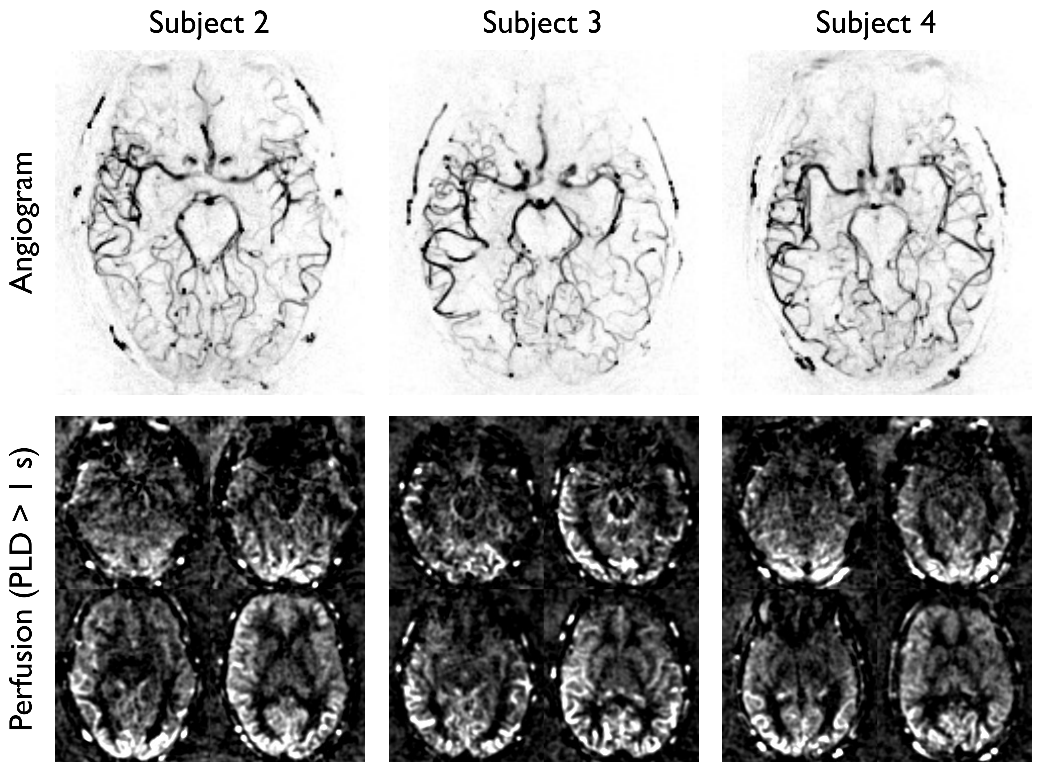

Supporting Figure S2: Temporal mean angiographic transverse MIPs and perfusion images with PLDs greater than 1 s for the other three subjects scanned in the first part of this study.
